# Research progress on biological functions of astragalus stems and leaves and their application in cattle production: a review

**DOI:** 10.1080/10495398.2026.2631838

**Published:** 2026-02-24

**Authors:** Chunfang Ma, Jinbao Zhang, Qi Yang, Qiaoe Zhang

**Affiliations:** a College of Animal Science and Technology, Ningxia University, Yinchuan, China; b Ningxia Hui Autonomous Region Veterinary Medicine and Feed Supervision Institute, Yinchuan, China; c Animal Husbandry and Aquatic Center of Yongning County, Yinchuan, China

**Keywords:** *Astragalus* stems and leaves, nutrients, active substances, biological functions, cattle production

## Abstract

*Astragalus* stems and leaves (ASL) represent a promising feed additive to improve cattle health and production efficiency. They are rich in nutrients and active substances, such as polysaccharides, flavonoids, alkaloids, exhibit antioxidant, antibacterial, anti-inflammatory, and other biological functions. With the advantages of high yield and low price, ASL are excellent potential feedstuff resources that could alleviate the shortage of conventional feed and reduce feeding costs. This review highlights the botanical characteristics, phytochemical components, nutritional and pharmacological effects of ASL, as well as their applications in cattle production, which could be beneficial for animal nutrition regulation and forage development.

## Introduction

Chinese herbal medicine, with a history spanning millennia, originates from natural sources such as plants, minerals, and animals. However, due to fluctuating prices and the limited availability of natural sources for these medicines, there is an increasing need to explore new herbal sources or utilize parts of traditionally used herbs that have previously been overlooked or underutilized.

The by-products of Chinese herbal medicine are characterized by abundant reserves, low production costs, and a low rate of comprehensive utilization. This not only results in significant waste of herbal resources but also leads to severe pollution of the ecological environment.^1^ According to incomplete statistics, there are currently approximately 4 million hectares of Chinese herbal medicines under artificial cultivation in China, and the amount of waste generated during the production of the medicinal parts of these herbs is about 60 to 70 million tons.^2^^,^^3^

It is considered a standard, natural, less toxic, and residue-free feed additive. Specifically, with the high content of nutrients and active substances, the by-products of Chinese herbal medicine can be rationally developed as feed. This could not only alleviate the shortage of feed raw materials in China, reducing feeding costs, but also mitigate the pressure of environmental pollution.

*Astragalus membranaceu* (AM) is a traditional Chinese herbal medicine belonging to the Leguminous plant family, with its medicinal part being the underground root. The yield of the above-ground part of AM is several times greater than that of the root. However, the stems and leaves of AM are discarded as waste in most Chinese production areas, which represents a serious waste of resources. Previous research indicates that ASL contain a significant amount of nutrients and active substances.^4–7^ The rational development of ASL as feed can not only alleviate the shortage of feed raw materials and reduce feeding costs in China but also mitigate environmental pollution pressures. ASL has been successfully utilized in livestock and poultry. Therefore, this paper reviews the botanical characteristics, phytochemical components, nutritional and pharmacological effects of ASL, and their application in cattle production, to provide a theoretical basis for the rational and scientific utilization of ASL.

## The botanical characteristics of AM

Due to these characteristics (see Table 1), AM has an unique position and wide application in Chinese herbal medicine.^8^^,^^9^

**Table 1.** Detailed table of botanical characteristics of AM.

Characteristic	Description
Scientific name	*Astragalus membranaceus*
Family	*Fabaceae*
Category	*Astragalus*
Distribution	It is mainly distributed in China, Mongolia, Korea and other places
Growing environment	Loves sunny, well-drained soil, cold and is tolerant to cold and drought
Morphological characteristics	Perennial herb, 30-90 cm tall, stems erect or obliquely ascending, multi-branched
Leaf	Pinnately compound leaves, 11-31 leaflets, elliptic or ovate, 1-2 cm long and 0.5-1 cm wide
Flower	Racemes, corolla butterfly, yellow or pale yellow, 6-8 months
Fruit	Pod, oblong, 2-3 cm long and 0.5-1 cm wide, containing multiple seeds
Root	The taproot is stout, cylindrical, yellowish-brown in surface, white in interior, with longitudinal wrinkles and transverse pores
Medicinal parts	Root
Harvest processing	Pick in autumn, wash, sun-dry or air-dry

## Nutrients, phytochemical and pharmacological effects of ASL

### Regular nutrients

Chinese herbal by-products are rich in nutrients. In practical application, it is necessary to determine the nutrient content of these by-products and adjust the additive amount according to the nutritional requirements of animals. The conventional nutritional components of Chinese herbal by-products include crude protein, crude fat, acid detergent fiber, neutral detergent fiber, as well as calcium and phosphorus, among others. As can be seen from Table 2, the crude protein content of Chinese herbal wastes ranges from 1.19% to 38.45%, crude fiber content ranges from 0.67% to 45%, crude fat content ranges from 0.11% to 12.3%, crude ash content ranges from 1.65% to 12.41%, and calcium and phosphorus contents range from 0.15% to 5.7% and 0.13% to 2.68%, respectively.^10–25^ Zhang et al.^5^ determined the nutrients of ASL, and the results showed that the contents of dry matter, crude protein, crude fiber, crude fat, calcium, and phosphorus were 92.5%, 11.7%, 37.2%, 1.6%, 1.5%, and 0.10%, respectively.

**Table 2.** Conventional nutrients of chinese herbal by-products (dry matter basis, unit: %).

Name	Dry matter	Crude protein	Coarse fiber	Crude fat	Crude ash	Calcium	Phosphorus	References
Stem and Leaves of Panax notoginseng	85.11-90.28	9.63-15.18	8.33-20.45	0.46-0.82	6.72-9.65	0.94-1.70	–	^10^
Stem and Leaf of Licorice	86.4	22.62	31.60	3.60	11.60	4.54	–	^11^
Stems and Leaves of pseudostellariae	–	13.39	8.32	2.57	3.23	0.95	0.53	^12^
Stem and Leaf of Astragalus	88.82-95.85	9.91-14.47	17.44-25.89	1.42-4.98	4.81-12.41	0.15-0.53	–	^13^
Stems and Leaves of Bupleurum	89.54	14.98	12.90	2.45	6.28	1.08	0.36	^14^
Stem and Leaf of Heracleum hemsleyanum	93.90	10.56	19.60	1.80	10.80	5.7	2.68	^15^
Stem and Leaf of Skullcap baicalensis	–	7.68		0.57	–	0.65	0.13	^16^
Stems and Leaves of Ginger	8.48	17.24	–	3.51	–	1.2	0.25	^17^
Stems and Leaves of Mulberry branch	89.82	14.5	32.11	1.32	10.87	–	–	^18^
Stems and Leaves of Peanut	90.48	8.34		1.90	11.53	1.43	0.14	^19^
Stems and Leaf meal of spearmint	87.4	12.8	29.5	1.4	7.3	–	–	^20^
Stem and Leaves of Isatis indigotica	94.5	25.16	8.2	2.7	11.0	–		^21^
Stem and Leaf of Curcuma ­­pha-eocaulis	36.54	7.61	–	5.8	10.65	0.16	0.13	^22^
Stem and Leaf of Tagetes erecta L	22.52	11.48	35.63	2.48	11.96	1.10	0.39	^23^
Stems and Leaves of Purslane	6.2-7.6	1.19-2.26	0.67-1.07	0.11-0.30	1.65-1.82	0.40-1.00		^24^
Stems and Leaves of Sweet Potato	–	9.35-38.45	35.3-45	1.36-12.30	–	–	–	^25^

From Table 2, it is evident that the crude protein content of ASL (11.7%) is at a moderate level among Chinese herbal wastes. In comparison to traditional roughage, such as corn stover (which typically has a crude protein content of about 4% to 6%), it exhibits certain advantages. Nevertheless, its crude fiber content (37.2%) is relatively high, which could impact its inclusion ratio and digestibility in cattle feed. Therefore, in future applications, additional attention and research are required to explore ways to enhance its utilization rate through suitable processing methods.

### Phytochemical of ASL

**Figure 1.**
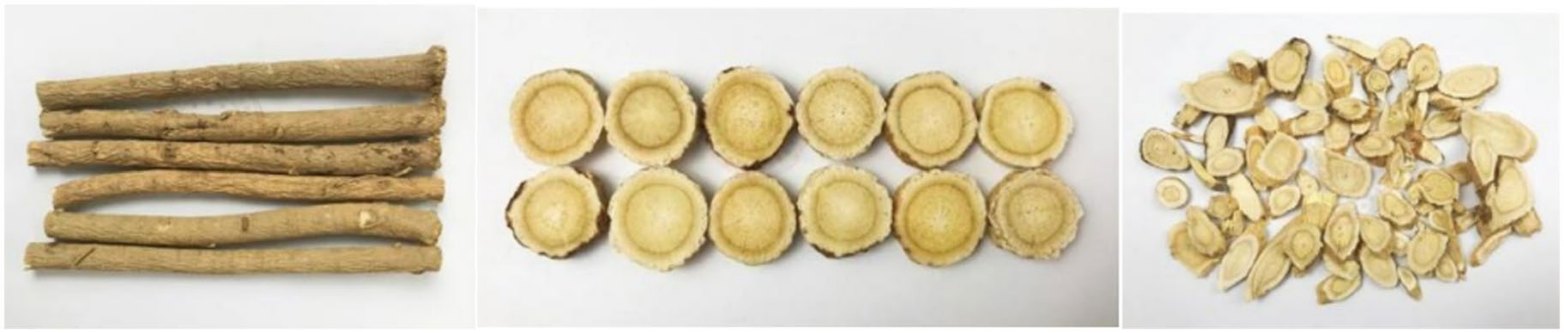
The roots of AM.

**Figure 2.**
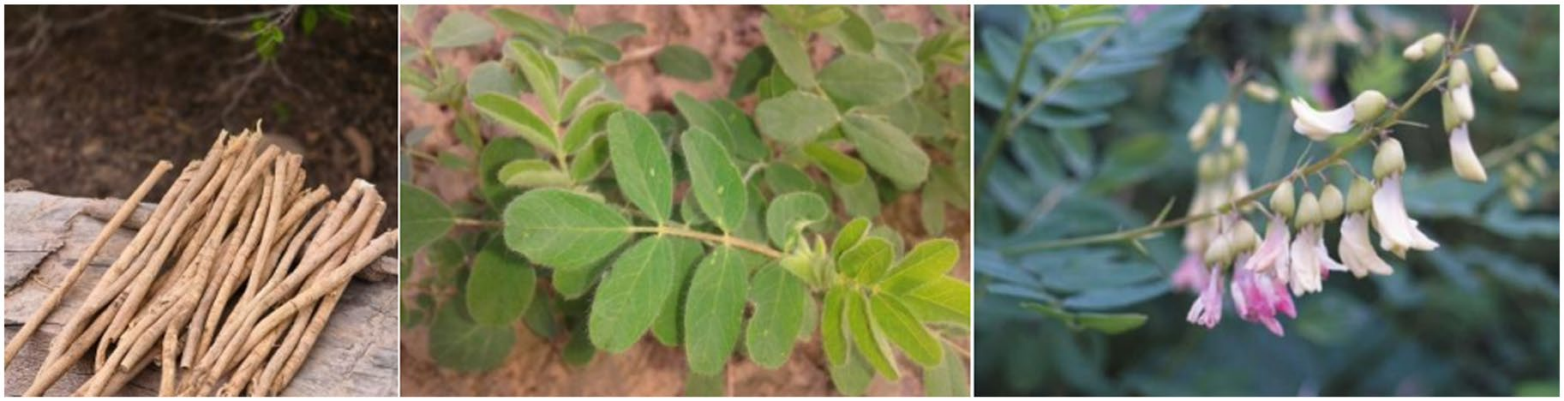
The stems, leaves and flowers of AM.

Most by-products of Chinese herbal medicine contain active substances such as polysaccharides, flavonoids, glycosides, alkaloids, and others, which can enhance the immunity and antioxidant capacity of animals.^26^ The findings from literature reports are as follows: Compared to radix *Astragalus* root(Fig. 1), ASL has a higher total flavonoid content, while the total saponins content is similar. The Astragaloside content in ASL is greater than that in the root, with the primary contribution coming from the *Astragalus* leaves (Fig. 2). However, the polysaccharide content is highest in the root, followed by the stems and leaves. The contents and types of bound amino acids and free amino acids are similar among *Astragalus* roots, stems, and leaves. ASL also contains various mineral elements, including major elements such as Na, K, Ca, and trace elements such as Fe, Cu, Zn.^27^ Zhang et al.^28^ demonstrated through experiments that the total saponin content in *Astragalus* leaves is 5-6 times greater than that in the roots, with the content in the stems being slightly lower than in the roots. Therefore, it is suggested to develop the above-ground parts of AM. Wang et al.^29^ determined the polysaccharide content in *Astragalus* flowers and stems using ultraviolet spectrophotometry, and the results indicated that the polysaccharide content in the stems (10.599 mg/g) is slightly higher than that in the flowers (2.676 mg/g). In *Astragalus* stems, the total flavonoids content is 1.621 mg/g, total saponins is 1.768 mg/g, and polysaccharides is 12.057 mg/g. In *Astragalus* flowers (Fig. 2), the total flavonoids content is 4.389 mg/g, total saponins is 21.842 mg/g, and polysaccharides is 11.213 mg/g. From the above data, it is evident that the content of various components in the flowers is higher than that in the stems, as determined by Zhu et al.^30^

In summy, the aboveground part of ASL has great utilization value. Additionally, ASL not only contain a variety of trace elements, but also include sterols, folic acid, linolenic acid, linoleic acid, betaine, bile Alkali, caffeic acid, cloronic acid, coumarin, niacin, riboflavin, vitamin P, starch, etc, therefore have great medicinal potential. Moreover, in ASL, active substances such as polysaccharides, flavonoids, and saponins do not act in isolation. For example, polysaccharides may enhance the absorption and utilization of flavonoids and saponins by the body through regulating the animal’s immune system, while flavonoids and saponins may cooperate with each other in the process of antioxidation and antibacterial action, forming a synergistic defense system to jointly maintain the health of the animal. However, the specific synergistic mechanism among these active substances is still not fully understood and requires further in-depth research.

#### Polysaccharides in ASL

**Figure 3.**
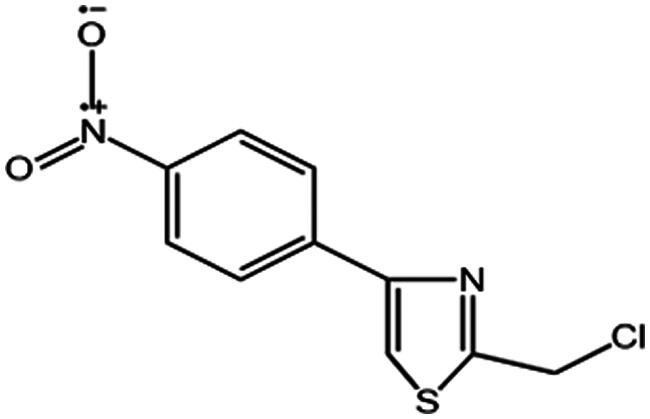
Structural formula of APS.

*Astragalus* polysaccharide (APS) is one of the primary active components of ASL, primarily consisting of glucose, fructose, and galacturonic acid. Its structural formula is depicted in Fig. 3. The high-purity form of APS is a fine, light yellow powder with a slightly sweet taste, moisture-retaining properties, and it offers anti-oxidative and immune-regulatory benefits.^31^

#### Flavonoids in ASL

**Figure 4.**
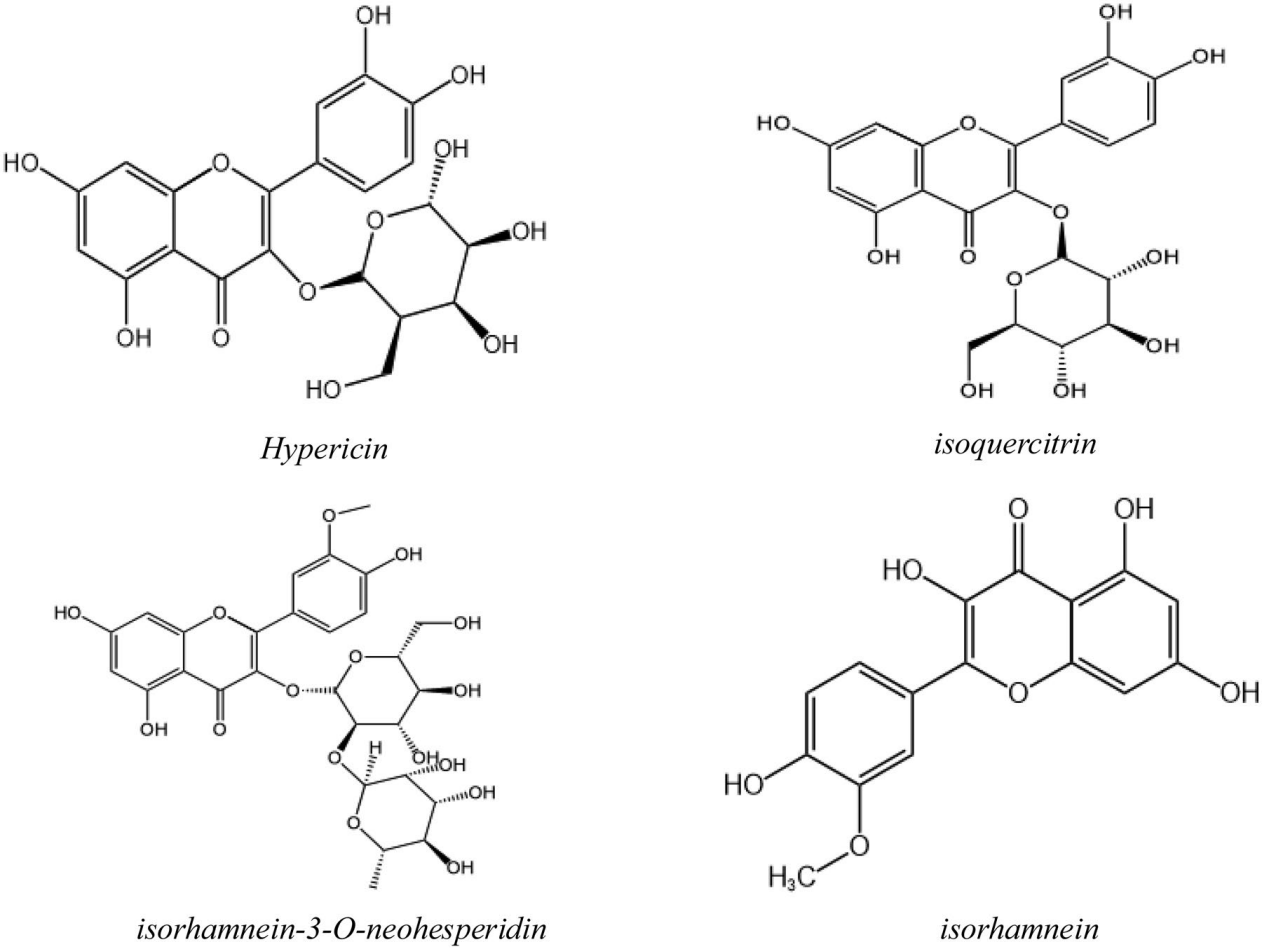
Structural formula of *hypericin, isoquercitrin, isorhamnein-3-O-neohesperidin* and *isorhamnein.*

Bi^32^ isolated the chemical constituents of flavonoids from the ASL, identifying five unique flavonoids: hypericin, isoquercetin, isorhamnetin-3-O-neohesperidoside, astragalin, and isorhamnetin. Their structural formulas are depicted in Fig. 4. These active ingredients possess bactericidal, anticancer, and immune-boosting properties.^33^

#### Saponins in ASL

**Figure 5.**
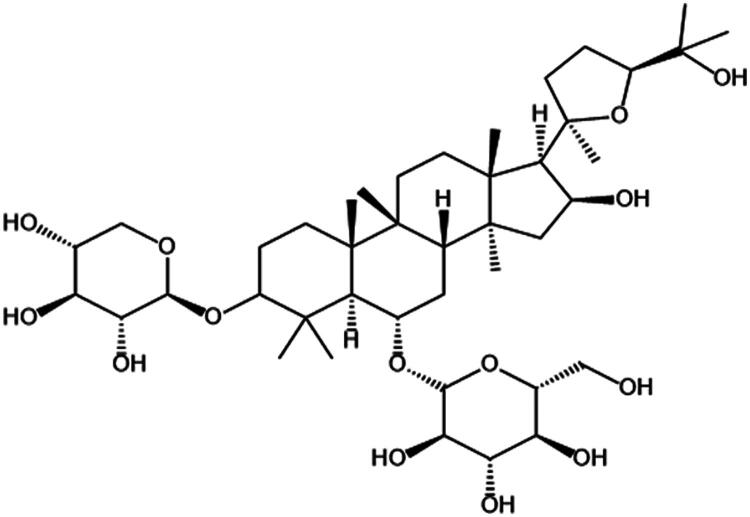
Structural formula of astragaloside.

The saponins present in ASL primarily consist of astragaloside, whose structural formula is depicted in Fig. 5. It is a white crystalline powder and serves as the key component for assessing the quality of ASL. Astragaloside exhibits anti-viral properties, liver protective effects, and heart-strengthening benefits.^34^

#### Alkaloids

**Figure 6.**
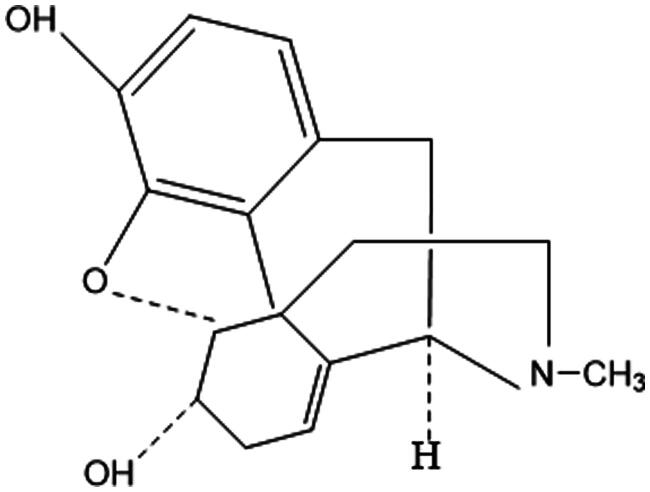
Structural formula of alkaloids.

Alkaloids are a class of nitrogen-containing, basic organic compounds. Their salts are soluble in water and ethanol but insoluble or sparingly soluble in organic solvents. The structural formula is depicted in Fig. 6. Alkaloids found in ASL exhibit anti-inflammatory and antioxidant properties, which can help reduce tissue damage.^35^

#### Amino acid

Amino acids are abundant in ASL, comprising a variety of essential amino acids such as glutamic acid, aspartic acid, and isoleucine, among others. As the fundamental building blocks of proteins, they are crucial for the growth, development, and normal functioning of both humans and animals.^36^

#### Other chemical composition

ASL contains a variety of essential amino acids and trace elements, including zinc and selenium, which play a crucial role in maintaining the body’s normal metabolism. Additionally, phenolic acids such as caffeic acid and chlorogenic acid possess antioxidant and anti-inflammatory properties.^37^

### Pharmacological effects of ASL

The chemical components in ASL are similar to those in the *Astragalus* root, and have similar pharmacological activities.

#### Anti-inflammatory and immune regulation

In the study by Chen,^38^ two homogeneous polysaccharides, *APS-A1* and *APS-B1*, were isolated from AM using *DEAE-52* cellulose and Sephadex *G-100* column chromatography. Bioactivity assays indicated that *APS-A1* and *APS-B1* possessed potential anti-inflammatory activity. The total flavonoids of AM have been shown to ameliorate lung fibrosis through inflammatory modulation and epithelium regeneration.^39^ The immunomodulatory effects of Astragali Radix are largely attributed to its active components, including polysaccharides, saponins, and flavonoids. In Chen’s research,^40^ the mouse lung cancer model employed is representative to some degree, as lung cancer mice typically exhibit a disordered immune system and an enhanced inflammatory response. By administering the isolated *APS-A1* and *APS-B1*, it was observed that within a certain dose range (e.g. [specific dose interval]), they could significantly enhance the immune organ index and serum cytokine levels of mice in a dose-dependent manner. This suggests that the polysaccharide components in ASL may exert anti-inflammatory and immunomodulatory effects by regulating specific immune cell subsets and cytokine networks. Nonetheless, the precise cellular and molecular mechanisms require further investigation through cell experiments and molecular biology techniques.

Li et al.^7^ demonstrated that *Astragalus* residue polysaccharide can enhance the immune organ index, the percentage of lymphocyte subsets in the spleen, and the level of serum cytokines in mice with lung cancer, indicating its immunomodulatory function. The ASL have been shown to improve the immunological functions of chickens, including the NDV antibody titers, the contents of *IFN-γ*, and *IL-2.*^41^ The immune-regulating properties of the flavonoids in ASL has been investigated, revealing their ability to promote the proliferation of lymphocytes induced by ConA, increase the total T cell count, regulate T cell subsets disturbances, and enhance the *LAK* activity induced by recombinant interleukin-2 (rIL-2).^42^ Additionally, AM has been found to modulate the intestinal immune response during sepsis by mediating the proliferation of *ILC3* through *RORγt.*^43^

#### Antioxidant capacity

*AM* is a rich source of polysaccharides that function as potent antioxidants. As a traditional Chinese herbal medicine, AM has been shown to enhance the growth, antioxidant, and immune performance of fish.^44^^,^^45^ Huang et al.^46^ investigated the extraction process of total flavonoids from ASL and their antioxidant activity, discovering that *Astragalus* exhibits a high scavenging ability for *DPPH* free radicals, *hydroxyl* free radicals, and superoxide anion free radicals. When compared with vitamin C at a concentration of 0.4 mg/mL, the ability of ASL (ASL) to eliminate these three types of free radicals was 96.09%, 89.75%, and 77.27% that of vitamin C, respectively. Yan et al.^47^ examined the effects of *Astragalus* extract on the growth performance, blood biochemistry, and antioxidant capacity of juvenile Carassius auratus. Their findings suggest that AM could be recommended for promoting growth and enhancing antioxidant capacity in aquaculture animals. The supplementation of broiler feed with AM powder at levels of 0, 100, 200, and 300 mg/kg diet increased the weight of immune organs, *IgG* levels, and improved liver and kidney functions and antioxidant status. Research into the impact of dietary Astragalus membranaceous supplementation on the oxidative stability of goat muscles has demonstrated that such supplementation significantly affects superoxide dismutase (SOD) and catalase (CAT) levels (p < .001).^48^

#### Antibacterial

Guo^49^ investigated the antibacterial activity of total saponins extracted from ASL, and the findings indicated that saponins exhibited a significantly superior antibacterial effect compared to flavonoids. Notably, saponins from ASL demonstrated a potent antibacterial effect against Escherichia coli, with a minimum inhibitory concentration (MIC) value of 12.5 μg/mL. Xue et al.^50^ examined the effects of APS on porcine circovirus type 2 (*PCV2*) infections both in vivo and in vitro, and discovered that APS suppresses *PCV2* infection by inhibiting endoplasmic reticulum stress. Astragaloside IV was shown to promote the polarization of the anti-inflammatory (M2) macrophage phenotype, thereby enhancing the deposition of extracellular matrix and the formation of blood vessels.^51^ Samuel, A. et al.’s study indicated that the polysaccharides found in AM possess antibacterial properties.^52^ Zhao et al.’s results also confirmed the enhancement of APS on LL-37 induction and antibacterial action in respiratory epithelial cells, which may be attributed to the activation of p38 MAPK/JNK and NF‐κB pathways. Furthermore, these results support the clinical application of APS in the treatment of infectious diseases.^53^ Kanaan et al.^54^ evaluated the antimicrobial and antibiofilm activity of extracts from AM against three Gram-positive bacterial strains. The results indicate that the whole plant ethanolic extract exhibited the highest bacteriostatic effect at a concentration of 12.78 mg/ml, and it also demonstrated the most versatile effect against the three different strains.^55^

#### Regulate blood sugar and lipids

Studies have indicated that APS can enhance glucose and lipid metabolism disorders, reduce inflammation and oxidative stress levels, and mitigate organ damage in *T2DM* mice.^56^ The research demonstrated that Astragaloside *IV* suppressed lipolysis by decreasing *cAMP* accumulation through the regulation of *Akt/PDE3B*, thereby contributing to the limitation of hepatic lipid deposition and the restraint of excessive hepatic glucose production.^57^

#### Anti diabetic activities

AM, a traditional Chinese medicine, exhibits a range of biological activities, including anti-diabetic effects. Its anti-diabetic mechanism primarily involves APS, which enhances the insulin signaling pathway and improves insulin sensitivity.^58^ It promotes glucose uptake and utilization by activating *AMPK* (AMP-activated protein kinase) and *PI3K/Akt* (Phosphoinositide 3-kinase/Protein kinase B) pathways.^59^ Flavonoids and polysaccharides possess significant antioxidant activity, which can reduce oxidative stress and protect islet beta cells. They also reduce lipid peroxidation by scavenging free radicals and improve diabetes-related oxidative damage.^60^ Furthermore, saponins and polysaccharides can inhibit the release of inflammatory factors, such as *TNF-α* and *IL-6*, by suppressing the *NF-κB* (Nuclear factor kappa-light-chain-enhancer of activated B cells) pathway, thereby reducing the inflammatory response. Additionally, the active ingredients in AM can inhibit the apoptosis of islet beta cells and promote their proliferation and functional recovery. By regulating the ratio of *Bcl-2/Bax*, the activity of caspase-3 is inhibited, thus reducing apoptosis.^61^

#### Other properties of ALS

Yu et al.^61^ demonstrated that APS could inhibit the proliferation of human gastric cancer *MGC-803* cells, release cytochrome C, activate the expression of cysteine aspartate protease-9/-3, and cleave polyadenosine diphosphoribose polymerase by increasing the pro-apoptotic/anti-apoptotic ratio. The accumulation of intracellular reactive oxygen species and the disruption of mitochondrial membrane potential induce apoptosis in cancer cells. APS can also synergize with anticancer drugs to enhance the efficacy of cancer treatment. APS inhibit pancreatic cancer progression by downregulating the *TLR4/NF-κB* signaling pathway.^38^ AM affects the fecal microbial composition of young hens, as determined by *16S rRNA* sequencing. The results indicated that the microbial composition of hens fed a diet supplemented with AM was greater than that of the control group.^62^ Astragaloside IV (AsIV), a key active component of Astragalus membranaceous, has been acknowledged for its potential cardiovascular protective properties.^63^ Cycloastragenol inhibits adipogenesis and fat accumulation in vitro and in vivo by activating the Hedgehog signaling pathway.^64^ Some studies have shown that APS has a protective effect on the heart.^65^

## Application of ASL in cattle production

AM active ingredients can enhance the immunity and antioxidant functions of cattle, alleviate heat stress, promote growth, and reduce the feed-to-gain ratio.

### Application in calf production

Li et al.^66^ investigated the impact of ASL on the growth and blood indices of Holstein calves and discovered that ASL effectively reduced morbidity and mortality rates by 20% and 3.6%, respectively. The findings indicated that ASL could strengthen the immune system of calves and decrease the incidence of diarrhea, making it an excellent feed additive. Li et al.^67^ supplemented the diet of calves with *Astragalus* saponin IV, and the results revealed that, in comparison to the control group, the average daily weight gain of the calves increased by 0.15 kg/d, the feed-to-gain ratio decreased by 3.2%, the serum total protein content rose by 5.24 g/L, and urobilin levels decreased by 19.8%, effectively alleviating heat stress in the calves and reducing the incidence by 20%. Yang et al.^68^ demonstrated that adding 10 mg of Astragalus saponin IV to the calves’ diet increased serum glutathione peroxidase activity by 21.2%, reduced malondialdehyde content by 20.8%, and significantly enhanced the calves’ antioxidant capacity.

Overall, the effects of ASL and saponins on calves are multifaceted and interrelated. The reduction in morbidity and mortality indicates that they enhance the overall resistance of calves, which is closely related to the improvement of immune function (such as increased serum total protein content and improved immune organ index). At the same time, the changes in antioxidant indicators (such as increased glutathione peroxidase activity and decreased malondialdehyde content) indicate that ASL and saponins can reduce oxidative stress in calves, providing a good internal environment for their healthy growth and thus promoting the improvement of growth performance (such as increased average daily gain and decreased feed conversion ratio). These results provide strong evidence for the application of ASL in calf breeding. However, the optimal application dose and method for different calf breeds still need to be further studied and determined.

### Effects on milk yield

Nie et al.^69^ supplemented the diet of dairy cows with an AM compound preparation. The average daily milk yield of the cows reached 30.88 kg/(head·d), Compared with the control group, the milk fat content increased by 0.19 percentage points. Additionally, the serum malondialdehyde content and catalase activity were reduced by 12.2% and 12.6%, respectively. The activities of alanine transaminase and creatine kinase were decreased by 9.5% and 9.3%, respectively, and the antioxidant performance and serum biochemical indexes of calves were improved.^66^ Zeng et al.^70^ administered an APS injection (30 mL/d) to heat-stressed cows, and the results indicated that the relative expression levels of 2-keto butyric acid and glycine in the serum of the cows were increased by 62.1% and 36.6% compared to the control group, while the relative expression level of fructose was decreased by 45.2%. This effectively improved the serum metabolic function of the cows. Zhou et al.^71^ demonstrated that ASL biological fermentation can promote milk yield in dairy cows and reduce the incidence of mastitis, providing an experimental basis for the application of Chinese medicine by-products in the field of dairy cows. Huang et al.^72^ studied the recovery, immune function, and breeding efficiency of postpartum dairy cows fed AM as a feed additive. The study revealed that feeding AM as a feed additive during the postpartum period had positive effects on wound recovery, immune function, endocrine regulation, and breeding efficiency. Furthermore, Zeng et al.^73^ investigated the effects of APS on the serum metabolism of dairy cows under heat stress. Their findings suggested that APS affects the serum hormones of heat-stressed dairy cows and regulates their metabolism through glucose metabolism and amino acid metabolism pathways.

The AM compound preparation may perform better in increasing milk yield and improving milk quality due to the synergistic effect of multiple components. In contrast, the APS injection focuses more on regulating the serum metabolic function of dairy cows, particularly the significant regulatory effect on key metabolites under heat stress conditions. In practical applications, farmers can choose the appropriate AM preparation based on the specific situation and breeding goals of their dairy cows. However, further research is needed on the long-term application effects and safety of these different preparations.

### Application in beef cattle production

Huang et al.^74^^,^^75^ supplemented the diet of beef cattle with various Chinese herbs, including codonopsis and Astragalus. Their findings indicated that the serum *IL-2* and *CD^4+^* levels in beef cattle were significantly elevated, and their immune system was also enhanced. Zhang et al.^76^ investigated the impact of the Jianpihua wet formula, which contains ingredients such as gypsum, cablin, *Astragalus*, tangerine peel, and wood fragrance, on heat-stressed beef cattle. They discovered that it promoted lipid anabolism in these animals. Liu et al.^77^ examined the influence of two groups of Chinese herbal additives on the meat quality of beef cattle. The primary components were gypsum, tabouli, *Astragalus*, tangerine peel, atractylodes, and Pueraria root. The results showed that these additives could significantly increase the intramuscular fat content of the beef longissimus dorsi muscle, making it tender and uniform. Yang et al.^78^ researched the prevention and treatment of transport stress in beef cattle using a custom formula (APS+Shenlingbaizhu powder) and found that it exhibited anti-stress properties and could moderate changes in body weight, body temperature, respiration, and heart rate before and after transportation.

Existing studies have shown that various Chinese herbal additives positively affect beef quality, although their mechanisms of action and effects differ. For instance, the inclusion of AM in Chinese herbal compound preparations can notably enhance the intramuscular fat content of the longissimus dorsi muscle in beef cattle, resulting in meat that is more compact and uniform. This is potentially due to the regulation of gene expression related to fat metabolism or hormone levels in the cattle. In contrast, a proprietary blend of APS and Shenlingbaizhu powder has proven effective in alleviating transport stress and maintaining the stability of beef quality. Nonetheless, the precise metabolic processes and long-term impacts of these Chinese herbal additives on beef cattle remain unclear. Further research is required, and concurrently, a standardized beef quality evaluation system should be developed to more accurately gauge the effects of various additives.

## Description of toxic effects

ASL holds potential as feed additives in ruminant farming. However, its potential toxic effects should not be overlooked, particularly with long-term, high-dose usage. The toxicity primarily affects the digestive system, manifesting as symptoms such as diarrhea, slowed rumen peristalsis, and reduced feed intake. This is because saponins (including those from Astragalus) can disrupt the microbial balance in the rumen, inhibit the activity of cellulolytic bacteria, and excessive flavonoids may irritate the intestinal mucosa, leading to inflammation.^79^^,^^80^ Alkaloids in AM, such as choline, may produce free radicals during liver metabolism, resulting in lipid peroxidation. Prolonged consumption can increase the liver’s metabolic load, potentially causing liver toxicity, elevated serum ALT or AST activity, and vacuolar degeneration of stem cells.^81^ Polysaccharide components may impose a burden on renal tubule reabsorption by increasing the glomerular filtration rate, leading to renal toxicity. Excessive polysaccharide intake can also result in feedback inhibition after over-activation of the immune system. The estrogen-like effects of flavonoids could interfere with the endocrine system, causing reproductive toxicity, such as reduced sperm motility in bulls and decreased pregnancy rates in cows.^82^ Reports indicate that the inclusion of stem and leaf powder in the diet should be less than 5% of dry matter, and the extract should not exceed 0.3%. Additionally, combining these with probiotics (such as yeast) can mitigate the negative effects of saponins on the rumen.^83^ Fermentation treatment can degrade over 40% of the anti-nutritional factors of saponins.^84^^,^^85^ Currently, there is a scarcity of comparative studies on the sensitivity of different beef cattle breeds to AM toxicity.

## Conclusion and future prospect

ASL are rich in nutrients and contain homologous active substances, making them potential feed resources. Although they have demonstrated significant application effects in cattle production, such as enhancing immunity, promoting growth, and improving production performance, there are still some issues to address. For instance, the stability and extraction efficiency of active ingredients need improvement, and the optimal application schemes for various cattle breeds and growth stages have not been established. It is essential to further investigate the impact of ASL active substances on cattle performance and meat quality, and to explore the regulatory mechanism of these substances on animal physiological functions through multidisciplinary collaboration involving genomics, proteomics, metabolomics, and transcriptomics. In summary, employing modern biotechnologies like gene editing and proteomics, we should deeply investigate the synthesis and regulatory mechanisms of active ingredients in ASL, aiming to increase their content and stability in plants. Secondly, conduct extensive field trials and breeding experiments to develop precise supplementation plans for different cattle breeds (e.g. Holstein cows, Simmental cattle) and growth stages (e.g. calf, fattening, lactation), including dosage, timing, and method of supplementation, and establish corresponding effect evaluation models. Finally, strengthen collaboration with feed processing and breeding enterprises to develop efficient and cost-effective ASL feed products and application technologies, promoting their widespread adoption in the livestock industry. It is anticipated that these research efforts will preliminarily resolve current issues and provide more scientific and effective guidance for the application of ASL in cattle production.
